# Effects of Collagen Crosslink Augmentation on Mechanism of Compressive Load Sharing in Intervertebral Discs

**DOI:** 10.1007/s40846-016-0207-z

**Published:** 2017-01-19

**Authors:** Thomas P. Hedman, Weng-Pin Chen, Leou-Chyr Lin, Hsiu-Jen Lin, Shih-Youeng Chuang

**Affiliations:** 1 Department of Biomedical Engineering, University of Kentucky, Lexington, KY USA; 2 Department of Mechanical Engineering, National Taipei University of Technology, Taipei, Taiwan, ROC; 3 Department of Orthopaedic Surgery, Tri-Service General Hospital, National Defense Medical Center, Taipei, Taiwan, ROC; 4 Department of Orthopaedic Surgery, Kang-Ning General Hospital, No.26, Ln. 420, Sec. 5, Chenggong Rd., Neihu Dist., Taipei, Taiwan, ROC

**Keywords:** Intervertebral disc, Crosslink augmentation, Compressive creep loading, Load sharing, Stress distribution

## Abstract

Exogenous crosslinking has been shown to have potential for treating disc degeneration and back pain due to its ability to increase the strength and toughness of the annulus fibrosus, increase intervertebral joint stability, decrease intradiscal pressure, and increase fluid flow through the disc. Some results imply that crosslink augmentation may also lead to changes in the compressive load sharing properties of the disc. The objective of the present study was to evaluate directional stress distribution changes of the disc following genipin crosslinking treatment. Bovine lumbar motion segments were randomly divided into control and crosslinked groups. Annular strains were determined from simultaneous deformation measurements at various time points during compressive creep testing. Four stress components of the annulus were then calculated according to the previously measured modulus data. Immediately after the application of a 750-N compressive load, mean axial and radial compressive stresses in the crosslinked group were twofold higher than control means. Conversely, mean lamellae-aligned and circumferential tensile stresses of the crosslinked discs were 8- and threefold lower, respectively, compared to control means. After 1-h creep loading, the two compressive mean stresses in both the control and genipin-crosslinked specimens increased approximately threefold from their initial 750-N-loaded values. The two tensile mean stresses in the crosslinked group remained lower than the respective levels of the control means after creep loading. A greater proportion of annular compressive load support under compressive creep loading, with a commensurate decrease in both tensile stresses and strains, was seen in the discs following exogenous crosslink augmentation.

## Introduction

Extracellular matrix (ECM) modification, including age-related tissue changes, can effect stress distributions and mechanisms of load sharing in intervertebral joints [1–3]. Intervertebral discs are considered to work synergistically with the posterior elements and ligamentous structures for providing passive restraint capabilities of the spinal motion segment [4]. Modification of the disc ECM could potentially result in changes to the passive load support mechanism of the spinal motion segment. In addition, it is well known that the cells in the disc respond to mechanical stimulation through mechanoreceptors that modulate cell–matrix and cell–cell interactions. Consequently, matrix modification could affect the mechanical stimulation of cells and the response of cells to physiological loading, which can lead to a further modification of the ECM [5–7]. Studies have demonstrated that the cell growth rate and ECM production may be affected by mechanical stimulation [8–10]. In animal in vivo studies, it was shown that static compressive loading in the disc (immobilization) may cause harmful responses such as disorganization of the annulus fibrosus, an increase in apoptosis, associated losses of cellularity, and changes in the regulation of either proteoglycan, type I, or type II collagen [11–14].

One of the primary roles of the spinal motion segment in humans is to withstand compressive loads. The nucleus pulposus (NP) is constrained radially by the annulus fibrosus (AF) and caudally and cranially by cartilaginous endplates. With an increase in compressive loading, there is an interaction between the NP and the AF as the NP bulges outwards and is constrained by the AF. The NP acts hydrostatically with a linear proportional increase in intradiscal pressure (IDP) corresponding to an increase in the magnitude of compressive load [15]. The increased pressurization of the NP with corresponding axial deformation and radial disc bulge produces multidirectional stresses in the annular lamellae. Assuming quasi-static equilibrium in the axial plane, the applied compressive load on the disc will be directly resisted by the AF axial compressive force and a force produced by the hydrostatic pressure in the NP. Also associated with compressive loading conditions, the disc internal pressure exerted by the NP tends to stretch the annular lamellae outwards, but the tensile properties of the lamellar collagen resist this stretch. Assuming quasi-static equilibrium in the transverse plane, the transverse force due to NP hydrostatic pressure is a function of the radial compressive properties of the AF and of the circumferential and lamellae-aligned tensile forces of the bulging AF.

In this study, the effect of exogenous crosslinking using genipin on the load sharing properties of intervertebral discs was investigated. Collagen crosslinks are known to have an important role in contributing to the mechanical strength of load-supporting tissues [16, 17]. Human discs have a higher concentration of mature crosslinks (enzymatically derived) than other tissues, which may be an indication of their demanding mechanical requirements [17, 18]. With disc aging and degeneration, total collagen crosslinking increases; the quantity of mature crosslinks is decreased or unchanged, while the quantity of age-related crosslinks (non-enzymatically initiated by glycation, i.e., pentosidine) is increased [18, 19]. These data show that a natural, endogenous crosslink augmentation mechanism exists in discs. This endogenous crosslinking may provide mechanical benefits to aging discs, which otherwise deteriorate due to mechanical or chemical degeneration. Notably, a lower level of pentosidine crosslinks was observed in severely degenerated discs [19]. The absence of these age-related crosslinks in severely degenerated discs may signal their importance in maintaining tissue integrity in aging discs. Likewise, disc crosslink augmentation may provide mechanical advantages towards resisting the ongoing process of disc matrix degradation.

Genipin is a gardenia fruit extract that is known to be an effective collagen crosslinker. It is thought to link amino acid groups in intramolecular, intermolecular, and intermicrofibrillar bonds [20]. Genipin crosslinking with the collagenous tissues has demonstrated enzymatic stability [21], subcutaneous biocompatibility [22], and relatively low cytotoxicity [23] and genotoxicity [24]. Genipin crosslinking has been suggested as a possible treatment for resisting disc degeneration and reducing low back pain due to its demonstrated capabilities to improve intervertebral joint stability [25–27], increase AF strength and toughness [28], and double nutritional flow through the disc [29]. Crosslink augmentation of the disc can be administered locally via micro-invasive surgery, considering the structural uniqueness of the disc as an isolated, confined space, where a single injection can be effective [30, 31]. The biocompatibility associated with direct use of genipin in the disc is considered outside of the scope of the present investigation. However, a recent animal study demonstrated that genipin reagent is able to react with a stab-injured disc in vivo (AF, NP, or both) without any obvious morbidity, where the exogenous crosslinking restored the disc integrity following a short-term injection treatment [32].

In a previous study investigating the exogenous crosslinking effects on IDP, it was noted that IDP was lower in genipin-crosslinked disc specimens under axial compressive load and that the drop in IDP caused by creep loading was decreased [33]. In seeming opposition, another work revealed that genipin crosslinking doubled fluid flow into and out of the NP following compressive creep loading and recovery [29]. To harmonize these results, it was postulated that crosslink augmentation causes material property changes that affect compressive load sharing mechanisms in the disc. To characterize the load sharing variations that result from exogenous crosslink augmentation, the present study models and analyzes stress distribution changes using in vitro measurements of disc deformation and the known material property changes [28]. The specific, directional (circumferential tension—CT, radial compression—RC, lamellae-aligned tension—AT, and axial compression—AC) material property changes associated with genipin crosslinking of isolated AF specimens were described in detail in a previous study [28]. In the present study, simultaneous intervertebral disc deformations were measured. Combined with the previously determined anisotropic material properties, these data enabled calculation of tissue stresses and exogenous-crosslinking-induced changes in load sharing. An experimental model under static creep loading was chosen to both be consistent with previous studies [29, 33] and provide an indication of load sharing changes at differing levels of disc fluid content.

To our knowledge, no previous studies have measured external radial bulging and internal disc deformations simultaneously. Intradiscal deformations have been measured previously using implanted beads and threads [34, 35]. Internal disc deformations have also been measured using monofilament nylon threads implanted into the disc in the anteroposterior direction, allowing intradiscal displacement to be traced by magnetic resonance images [36]. Both of these techniques may introduce errors in the form of unknown movement between the implanted materials and annular lamellae. In the present study, two zero-contact methods were developed to quantify internal and external disc deformations in the transverse plane: a sonographic imaging technique directly measured internal deformation of AF, while a custom rotating laser measurement system simultaneously quantified disc bulge. Limited access to the sonographic equipment used in this study (one day) limited the number of total experiments conducted to eight.

## Materials and Methods

A total of eight bovine lumbar motion segments (4- to 6-month-old) were used in this study. Specimen preparation included cutting the pedicles and removing the posterior processes and the soft tissue around the disc. Great care was taken to maintain disc hydration using a saline mister at regular intervals. The specimens were randomly treated by either soaking in phosphate-buffered saline (PBS) solution as a control group (n = 4), or soaking in 0.33% (g/g) genipin (98% pure, Challenge Bioproducts Co., Taichung, Taiwan) plus PBS solution as a crosslinked group (n = 4). The specimens were soaked for two days at room temperature in their respective solutions before being tested. Soaking was used rather than injections of reagent in order to minimize variation of treatment coverage, which could potentially mask the treatment effects. A previous study verified that soaking treatment does not affect disc tissue hydration [33].

After individual treatment, the superior and inferior vertebrae of the motion segment specimens were potted in polyurethane for mechanical testing. An axial compressive load of 75 N was applied to the specimens by an Instron 8521 material testing system (Instron Corp., Canton, MA, USA) and held for 10 min for preconditioning. Continuously, the specimens were loaded by an axial compressive load of 750 N at a loading rate of 25 N/s, and then held at a constant load for a 1-h creep period. Disc height was simultaneously recorded by the Instron machine with a data acquisition rate of 10 Hz.

**Fig. 1:**
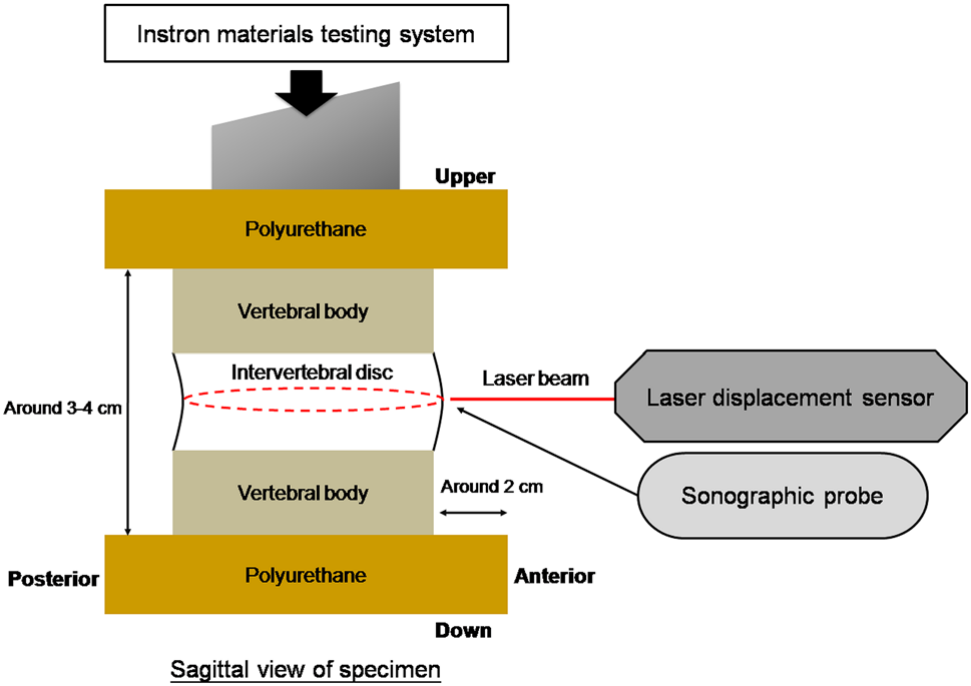
Test setup. Specimen was loaded using an Instron 8521 system mounted with a non-contact laser measurement system on a platform that rotates around the specimen. The *dotted circle* near the intervertebral disc represented the track of laser measurement on the circumferential surface of the disc. A 7.5-MHz hand-held sonographic probe was used to collect a simultaneous image of internal deformations

The external surface contour of the mid-transverse plane of the disc and the internal deformation of the AF were measured at three time points: (1) immediately after the 75-N compressive load was applied, (2) immediately after the 750-N compressive load was applied, and (3) after 1 h of creep loading but before ramp unloading. The external surface contour of the disc in the transverse plane was measured by a custom non-contact measurement system containing a rotating laser displacement sensor (LK-081, Keyence Corp., Elmwood Park, NJ, USA) (Fig. 1) with a resolution of 3 μm and a system accuracy of 0.01 mm. The measurement was started each time at the same disc location, marked by a colored marker at the mid-axial level on the disc surface, where the largest expected bulging would occur. The rate of data acquisition was 20 Hz, with 800 data points for a 360° complete circle. Circumference length and radius of the disc were calculated using custom-designed software based on the Matlab (MathWorks, Natick, MA, USA) programming environment. The radius data were further used for calculation of the lamellae-aligned tensile strain. Quasi-static assumptions allowed the circumferential tensile strain to be calculated as follows:

$$\varepsilon_{\text{CTx}} { = }\frac{{C_{\text{x}} {\text{- }}C_{0} }}{{C_{0} }}$$

where *ε*_*CT*x_ is the circumferential tensile strain, *C*_0_ is the contour length at a 75-N applied load, *C*_1_ is the circumferential length measured immediately after application of 750 N, and *C*_2_ is the circumferential length under load after 1 h of creep.

**Fig. 2:**
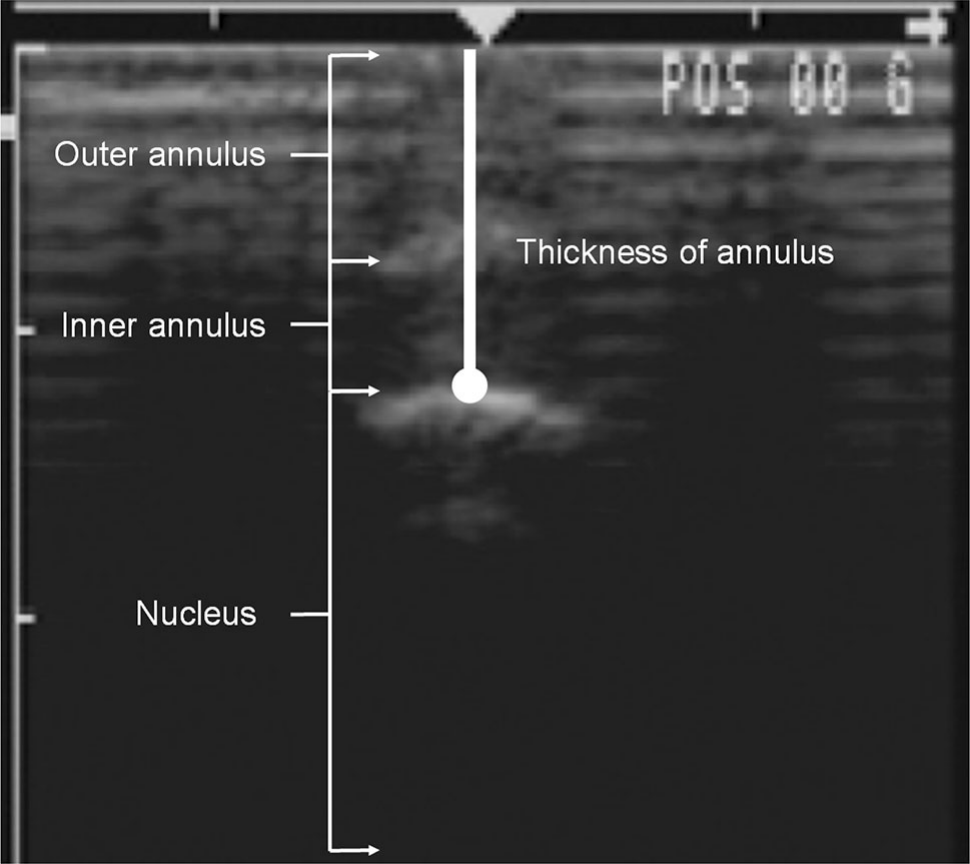
The thickness of annulus fibrosus was measured in sonographic images at different stages of compressive loading. The *left* border of the image is marked in centimeters

The internal deformation of disc was measured by an ultrasound imaging machine (280 SL, Johnson & Johnson Professionals, Inc., Ramsey, NJ, USA) in brightness mode with a 7.5-MHz linear array probe. The probe was applied to the anterior surface of the disc. The width between the outermost and innermost aspect of AF was measured using the built-in “edge (position) reader” of the sonographic machine (Fig. 2). The standard error of the measurements was 0.3 mm. The radial compressive strains were then calculated using the following equation:

$$\varepsilon_{\text{RCx}} { = }\frac{{S_{\text{x}} {\text{- }}S_{0} }}{{S_{0} }}$$

where *ε*_*RC*x_ is the radial compressive strain, *S*_0_ is the measured width with a 75-N load, *S*_1_ is the width at the onset of 750-N loading, and *S*_2_ is the width after 1 h of creep but prior to unloading.

The axial compressive strain was calculated based on the disc height data acquired by the Instron machine (±0.01 mm) using the following equation:

$$\varepsilon_{\text{ACx}} { = }\frac{{{{DH}}_{\text{x}} {{- DH}}_{0} }}{{{{DH}}_{0} }}$$

where *ε*_*AC*x_ is the axial compressive strain, *DH*_0_ is the disc height at a 75-N load, *DH*_1_ is the disc height measured immediately after applying 750 N, and *DH*_2_ is the disc height after 1 h of creep.

The lamellae-aligned tensile deformations under compressive load were approximated using circumferential bulging and disc height measurements. The lamellae-aligned tensile strain was calculated as the radial expansion minus the bulk expansion:

$$\varepsilon_{\text{ATx}} { = }\frac{{(R_{\text{x}} {\text{- }}R_{0} )\;{{- PE}}_{\text{x}} }}{{{{DH}}_{0} }}$$

where *ε*_*AT*x_ is the lamellae-aligned tensile strain, *R*_0_ is the radius at a 75-N load, *R*_1_ is the disc radius measured immediately after applying 750 N, and *R*_2_ is the radius after 1 h of creep. *PE* is the bulk expansion in the radial direction based on an equal, assumed Poisson’s ratio of 0.4, and the measured axial deformation.

Mean differences between treated and untreated strain measurements were compared using Student’s *t* test. Due to the aforementioned single-day restricted access to the sonographic equipment and an expected level of variability in the data, statistically significant differences were unlikely; consequently, the data trends were also estimated via 95% confidence intervals (CIs) of the strain differences between treated specimens and controls.

## Results

Radial compressive strain (internal deformation) measurements taken from sonographic images, immediately after 750-N axial loading, were 2.4 ± 0.8 and 4.5 ± 2.2% (mean and standard deviation, *p* = 0.123, 95% CI of difference: −4.96 to 0.76%) in the control and genipin groups, respectively. After 1 h of creep loading, the strains were 7.3 ± 2.4 and 14.4 ± 8.2% (*p* = 0.148, 95% CI of difference: −17.55 to 3.35%) in control and genipin groups, respectively. Axial compressive strains were 13.41 ± 1.79 and 11.06 ± 1.16% (*p* = 0.078, 95% CI of difference: −0.35 to 4.95%) in control and genipin groups, respectively, when initially loaded to 750 N. After 1 h of creep loading, the respective strains in control and genipin groups were respectively 36.47 ± 1.46 and 32.48 ± 3.65% (*p* = 0.092, 95% CI of difference: −0.89 to 8.89%). Circumferential strains associated with disc bulge were 2.41 ± 1.18 and 0.54 ± 1.60% (*p* = 0.106, 95% CI of difference: −0.55 to 4.35%) in control and genipin groups, respectively, under 750-N load. The circumferential strains increased to 3.90 ± 2.84 and 2.60 ± 0.99% (*p* = 0.416, 95% CI of difference: −2.34 to 4.94%) in control and genipin groups, respectively, after 1 h of 750-N creep loading. Lamellae-aligned tensile strains, immediately after 750-N axial loading, were calculated to be 8.7 ± 4.0 and 1.1 ± 7.2% (*p* = 0.115, 95% CI of difference: −2.48 to 17.68%) in the control and genipin groups, respectively. After 1 h of creep loading, these respective strains increased to 13.4 ± 13.0 and 8.5 ± 3.7% (*p* = 0.496, 95% CI of difference: −11.64 to 21.44%).

Utilizing modulus data from a previous study [28] and assuming a linear Hooke’s relationship between stress and strain, mean tissue stresses were calculated. The results are shown in Table 1. Compared to controls, mean axial compressive stresses were approximately doubled in the genipin group for both static and 1-h creep loading of 750-N magnitude (187 and 266%, respectively). A similar treatment-induced doubling occurred in the mean radial compressive stresses for both initial loading and after 1 h of creep loading (200 and 222%, respectively). Mean axial compressive stresses increased approximately threefold after 1 h of creep loading compared to stresses at the initial application of a 750-N load for both control and genipin groups (272 and 386%, respectively). A similar threefold increase due to creep loading was seen in the mean radial compressive stresses (300% for controls, 333% for genipin crosslinking). Mean tensile stress increases due to creep loading were 64% for circumferential and 56% for lamellae-aligned tension in the controls. Much larger mean tensile stress increases due to creep loading were seen in genipin-treated specimens, 385 and 800% for circumferential and lamellae-aligned tensile stresses, respectively. Yet, at the end of 1 h of compressive creep loading, the genipin-crosslinked discs experienced similar stress (18% less in circumferential tension and 28% less in lamellae-aligned tension) compared with that of controls on average. Under transient loading conditions, mean tensile stresses in the crosslinked discs were 3.5 and 8 times less than those of controls in circumferential and lamellae-aligned tension, respectively.

**Table 1:** Stress distributions in annulus fibrosus of control and genipin groups under compressive creep loading

Compressive properties	AC			RC		
	Initial	Creep	Creep/initial (%)	Initial	Creep	Creep/initial (%)
Control (MPa)	0.46	1.25	272%	0.03	0.09	300%
Genipin (MPa)	0.86	3.32	386%	0.06	0.20	333%
Genipin/control (%)	187%	266%		200%	222%	

Tensile properties	CT			AT		
	Initial	Creep	Creep/initial (%)	Initial	Creep	Creep/initial (%)
Control (MPa)	0.47	0.77	164%	0.16	0.25	156%
Genipin (MPa)	0.13	0.63	485%	0.02	0.18	900%
Genipin/control (%)	28%	82%		13%	72%	

Mean values are given. Stress unit: MPa

*AC* axial compression, *RC* radial compression, *CT* circumferential tension, *AT* lamellae-aligned tension

Initial stress represents the stress difference generated during the process of ramping from a load of 75 N to 750 N. Creep stress represents the stress generated from the 75-N load to 1 h of creep at 750 N. Tensile stresses were decreased in the genipin-crosslinked discs while compressive stresses were increased

## Discussion

Simultaneous non-contact radial disc bulge, disc height, and internal disc deformation measurements were made on bovine intervertebral discs subjected to sustained compressive loading of intact motion segments. Combined with the material properties previously measured from isolated AF specimens [28], these data enabled an analysis of load sharing changes brought about by genipin crosslink augmentation.

One observation from these data is the substantial reduction of the tensile strain associated with disc bulging with crosslink augmentation. Although not established beyond the condition of compressive creep loading used in the present study, reduction of disc bulging under load could have direct implications on neural compression and pain in the symptomatic disc. This parameter should be further evaluated under additional pseudo-physiologic loading profiles, including compression with bending. Nonetheless, these strain observations suggest that exogenous crosslinking treatment for the disc could potentially have benefits in regard to pain relief.

In the present study, it was found that average axial and radial compressive stresses of the annulus increased approximately two-fold due to crosslink augmentation at the onset of a 750-N load. Conversely, average lamellae-aligned and circumferential tensile stresses were decreased up to eight- and three-fold, respectively, due to exogenous crosslinking. Over 1 h of creep loading, the two compressive stresses in both control and genipin-crosslinked groups increased at comparable rates based on a percentage of initial stress. The two tensile stresses in the genipin-crosslinked group approached but did not reach the respective levels of the control means over the period of creep loading. These results suggest that the type of compressive load support was transformed from resembling a fluid pressurized, axially loaded elastic container to resembling a thick-walled, axially loaded elastic cylinder. In both static and creep loading conditions, a greater proportion of external compressive load was sustained by compressive stresses in the AF of the crosslinked discs compared to the control group. A previous study of crosslink augmentation effects on disc material properties demonstrated significant increases in circumferential and lamellae-aligned tensile yield strength, ultimate strength, and toughness [28]. Combined with the observed drop in tensile stress in the present study, these results bode well for the disc’s demanding mechanical environment in view of the propensity for tensile overload of posterior annular tissue when subjected to flexion-bending, as described by numerous authors [37–39]; however, the present study did not include bending loads.

This drop in tensile loading and increase in compressive loading in the AF corresponds with previous IDP results, which showed an initially lower IDP with a 750-N compressive creep load in the genipin group as compared to the control group [33]. Likewise, crosslinking reduced the loss of IDP (disc depressurization) during compressive creep loading [33]. In another set of experiments investigating hydration changes in different disc regions under a given compressive creep loading, it was demonstrated that outward fluid flow through the disc doubled in the genipin group as compared to that in the control group [29]. One would expect that greater fluid transport would correspond to accelerated loss of hydrostatic load support rather than the observed maintenance of IDP. The results presented in the present study suggest that the flow-induced drop in IDP may be moderated by the increased proportion of annular compressive load support associated with increased crosslinking.

Intervertebral disc degeneration involves a change of tensile and compressive properties of the AF, which, in turn, could influence the annulus fatigue resistance properties [40–43], and the propensity for pain-related failures of the disc such as bulges or herniations that impinge or compress adjacent neural tissues. Tensile strength and stiffness of the AF decrease with aging and degeneration [40, 44, 45]. Loss of tensile strength may lead to demand on the AF to support an increasing amount of axial compressive stress, which could further deteriorate the material properties of the AF, leading to a vicious cycle of degeneration that could lead to disc collapse and low back pain. Crosslink augmentation increases AF lamellae-aligned and circumferential tensile moduli and strength (yield strength increases of 45 and 78%, respectively) [28], while changing the mechanism of compressive load support according to the findings of the present study. Thus, one could suggest that crosslink augmentation may work towards preventing potential annular failures associated with disc bulges and herniations in moderately degenerated discs by both reducing lamellae-aligned and circumferential tensile stresses while dramatically increasing tensile strength [28].

To our knowledge, the technique used to measure internal disc radial compressive deformations using ultrasound equipment has not been reported previously. The standard deviation of repeated measurements was approximately 0.3 mm, which is more than the mean difference of radial compressive strain between crosslinked and control groups at initial loading and less than the mean difference after creep loading. The determination of the margin between the NP and the AF was made via visual observation by one trained observer, which likely contributed towards the measurement error. Care was taken to place the probe position and angle similarly with successive measurements, but probe position is also a potential source of errors. Various techniques could be employed to minimize measurement errors, including digital edge detection of the margin between NP and inner AF, and the design of an apparatus to hold the sonographic probe and register the position and angle relative to the specimen. Similarly, the lamellae-aligned tensile deformation measurement can be further refined by developing a method to directly measure this deformation. Several methods were attempted such as measuring the changes of the contours of the disc under load from digital images, tracing the lateral bulging point-to-point on the surface with monofilament nylon, and analyzing the contours of press-molds obtained from applying molding material to the disc surface. All of these measurement techniques provided inadequate repeatability or accuracy. Relying instead on the considerably more accurate measurements of circumferential bulging and disc height, lamellae-aligned tensile strain was approximated as radial expansion minus bulk expansion based on measured axial deformation and using an equal, assigned Poisson’s ratio of 0.4.

Caution should be exercised in making inferences about the mechanical effects of age-related increases in crosslinking of intervertebral discs based on the results of this study. Tensile strength and stiffness are known to decrease with aging and degeneration, at the same time that crosslinking generally increases. This may suggest that the opposing effects of endogenous crosslink augmentation are generally not sufficient to compensate for the declining mechanical properties of the matrix. To our knowledge, no data has been published that demonstrates the isolated effects of age-related crosslinks on AF yield strength, ultimate strength, and toughness. It is also interesting that Duance et al. found that the most severely degenerated discs had lower quantities of age-related crosslinks [19]. Future studies should analyze the effects of endogenous, age-related crosslinking on AF material properties and load sharing behavior in order to determine the extent to which endogenous crosslinking is beneficial to the mechanical integrity of aging disc tissue.

## Conclusion

This study demonstrated that the exogenous crosslinking could change the compressive load sharing mechanism of intervertebral disc. Under the given static and creep compressive loading, the tensile stresses in the disc were decreased while the compressive stresses were increased correspondingly following genipin treatment. As the tensile properties of AF were known important for maintaining disc integrity during disc bulging outwards, these results have implication that crosslink augmentation may be advantageous for the degraded or degenerated disc which has insufficient mechanical performance.
